# Improved anti-tumor immunity and efficacy upon combination of the IDO1 inhibitor GDC-0919 with anti-PD-l1 blockade versus anti-PD-l1 alone in preclinical tumor models

**DOI:** 10.1186/2051-1426-3-S2-P303

**Published:** 2015-11-04

**Authors:** Jessica Spahn, Jing Peng, Edward Lorenzana, David Kan, Thomas Hunsaker, Ehud Segal, Mario Mautino, Erik Brincks, Andrea Pirzkall, Sean Kelley, Sami Mahrus, Liling Liu, Stephanie Dale, Cristine Quiason, Elizabeth Jones, Yichin Liu, Sheerin Latham, Laurent Salphati, Kevin DeMent, Mark Merchant, Georgia Hatzivassiliou

**Affiliations:** 1 Genentech, South San Francisco, CA, USA; 2 NewLink Genetics, Inc., Ames, IA, USA

## 

Indoleamine 2, 3-dioxygenase (IDO1) is a cytosolic enzyme catalyzing the oxidation of tryptophan to kynurenine. IDO1 plays an important role in modulating immune responses in cancer, where increasing evidence suggests that IDO1 expression by dendritic cells (DCs) and other antigen-presenting cells (APCs) contributes to tumor-mediated immune suppression. Programmed death-ligand 1 (PD-L1) is a cell surface protein that is broadly expressed by tumor cells and tumor-infiltrating immune cells in many human cancers. Blockade of PD-L1 or PD-1 with monoclonal antibodies results in strong and often rapid anti-tumor effects in preclinical models and in the clinic, where deep and durable objective responses have been observed in a number of tumor types including melanoma, NSCLC, renal and bladder cancer. Accumulating preclinical and clinical data provide evidence that host immunosuppression by tumor cells is mediated by multiple pathways, and therefore combination therapy regimens employing one or more targeted immunotherapy agents are necessary for more complete and durable patient responses. The combination of IDO1 inhibition with PD-L1 blockade has strong rationale since both checkpoints are co-expressed in many cancers and inhibit immune responses via complementary mechanisms. Using mouse syngeneic tumor models we present further evidence of the therapeutic potential of combining IDO1 inhibition with anti-PD-L1 blockade. We observe improved depth and duration of responses in mice treated with the combination regimen versus anti-PD-L1 blockade alone. These studies also reveal potential immunomodulatory effects responsible for the observed efficacy, namely an improved CD8:Treg ratio and evidence of increased maturation and antigen presentation capacity by DCs and APCs in the combination arm. Clinical trials will further assess the potential of this promising combination regimen in patients. A Phase Ib, open-label, multicenter, global study is underway to evaluate the safety, tolerability, and pharmacokinetics of the combination of GDC-0919 and atezolizumab (MPDL3280A, anti-PD-L1) in patients with locally advanced or metastatic incurable solid tumors (NCT02471846).

